# Induced Pluripotent Stem Cells to Model Juvenile Myelomonocytic Leukemia: New Perspectives for Preclinical Research

**DOI:** 10.3390/cells10092335

**Published:** 2021-09-06

**Authors:** Zeinab Wehbe, Foued Ghanjati, Christian Flotho

**Affiliations:** Division of Pediatric Hematology and Oncology, Department of Pediatrics and Adolescent Medicine, Medical Center, Faculty of Medicine, University of Freiburg, 79106 Freiburg, Germany; zeinab.wehbe@uniklinik-freiburg.de (Z.W.); foued.ghanjati@uniklinik-freiburg.de (F.G.)

**Keywords:** JMML, leukemia, reprogramming, IPSC, differentiation, hematopoietic cells

## Abstract

Juvenile myelomonocytic leukemia (JMML) is a malignant myeloproliferative disorder arising in infants and young children. The origin of this neoplasm is attributed to an early deregulation of the Ras signaling pathway in multipotent hematopoietic stem/progenitor cells. Since JMML is notoriously refractory to conventional cytostatic therapy, allogeneic hematopoietic stem cell transplantation remains the mainstay of curative therapy for most cases. However, alternative therapeutic approaches with small epigenetic molecules have recently entered the stage and show surprising efficacy at least in specific subsets of patients. Hence, the establishment of preclinical models to test novel agents is a priority. Induced pluripotent stem cells (IPSCs) offer an opportunity to imitate JMML ex vivo, after attempts to generate immortalized cell lines from primary JMML material have largely failed in the past. Several research groups have previously generated patient-derived JMML IPSCs and successfully differentiated these into myeloid cells with extensive phenotypic similarities to primary JMML cells. With infinite self-renewal and the capability to differentiate into multiple cell types, JMML IPSCs are a promising resource to advance the development of treatment modalities targeting specific vulnerabilities. This review discusses current reprogramming techniques for JMML stem/progenitor cells, related clinical applications, and the challenges involved.

## 1. Introduction

Juvenile myelomonocytic leukemia (JMML) is a myeloproliferative neoplasm mainly affecting infants and young children [1,2,3,4,5]. The disorder results from constitutive genetic activation of the Ras signal transduction pathway, leading to excessive formation of differentiating leukemic cells along the myelomonocytic and red cell lineage [1,6,7]. The clinical presentation and course of JMML is heterogeneous and strongly depends on the particular Ras pathway driver gene [6,8,9]. Driver mutations in one or more genes interfering with Ras signaling occur in a monoallelic form in the germline (*NF1* or *CBL;* almost always accompanied by secondary somatic loss of heterozygosity in leukemic cells) or as a monoallelic somatic lesion acquired in hematopoietic cells (*PTPN11*, *NRAS* or *KRAS*) [1]. Secondary genetic alterations can also occur including mutational events in the *EZH2*, *ASXL1*, *SETBP1*, or *JAK3* genes [1,8,10,11]. In addition to the genetic profiling, JMML can be classified based on changes in DNA methylation [1,3,5]. Epigenetic treatment using the DNA methyltransferase-inhibiting agent azacitidine has now emerged as an important part of the therapeutic arsenal [12,13,14]. However, approximately one-third of JMML cases run a fatal course despite azacitidine and/or allogeneic hematopoietic stem cell transplantation (HSCT) [9]. Consequently, improving the treatment options for JMML remains a necessity. A major limiting factor for in-depth studies of JMML pathobiology is its rare incidence (1 to 9/1,000,000 based on orphanet) [15], making it difficult to collect large amounts of primary research material. In addition, unlike acute leukemia, JMML cell populations do not exhibit a maturation arrest [16], which makes it almost impossible to generate stable immortalized cell lines. Long-term maintenance of JMML cells in culture is hampered by rapid differentiation and apoptotic behavior [17]. Over the past two decades, human induced pluripotent stem cells (IPSCs) have rapidly become a valuable source for safely generating highly differentiated cells in unlimited quantities for basic science, as well for diverse clinical applications. Hence, IPSCs hold the promise to be a realistic alternative to overcome all of these limitations. It is encouraging that five research studies have already succeeded in generating IPSCs from JMML cells and differentiating these into hematopoietic cells [18,19,20,21,22]. The added value of IPSC technology for studying JMML pathogenesis and its potential as drug development platform will be the focus of this feature paper.

## 2. Previous Strategies of Modeling JMML

### 2.1. In Vitro Approaches

Maintaining patient-derived primary cells in a dish is appealing to every researcher. Starting as early as 1974, cells derived from bone marrow (BM) and peripheral blood (PB) of patients diagnosed with JMML were cultured in semisolid media. The experiments uncovered a characteristic capacity of JMML progenitor cells for excessive formation of monocyte-macrophage colonies in vitro [23]. This feature was repeatedly observed in the context of different studies [24,25,26,27]. Cultures in semisolid media were also instrumental in elucidating the hypersensitive response of JMML progenitor cells to several hematopoietic cytokines, specifically the granulocyte-macrophage colony-stimulating factor (GM-CSF) [28,29], tumor necrosis factor α [30], and interleukin-1 [31]. Other studies were geared toward inhibiting the proliferation and the colony formation of JMML cells, with the ultimate goal to find a potential therapy for the disease [32,33,34,35]. For example, the inhibition of the above factors by interleukin-10 resulted in decreased colony formation and cell viability [36].

Immortalized cells that have been manipulated to proliferate indefinitely may also be envisioned as a potential source for modeling JMML. The generation of such cell lines requires ectopic expression of oncogenes, telomerase reverse transcriptase expression, and/or inactivation of tumor suppressor genes [37,38]. Keeping immature JMML progenitor cells in culture poses a big challenge, as they tend to differentiate and undergo rapid senescence [17]. At least, it is possible to maintain undifferentiated JMML cells for approximately 2 weeks in medium supplemented with stem cell factor, Fms-like tyrosine kinase 3 ligand, thrombopoietin (TPO), and interleukin-6, which is sufficient for viral transduction and reprogramming (unpublished own observations). The difficulties are illustrated by the fact that not a single JMML cell line is included in a repertoire of 100+ leukemia cell lines curated in a large academic collection of microorganisms and cell cultures [39]. Traditionally, it is seen as an advantage of immortalized cell lines that they can be characterized and standardized thoroughly, facilitating the comparison of discoveries made in different labs and enhancing interaction between researchers. However, recent studies have highlighted issues with uniformity and reproducibility in cell lines after manipulation and long-term passaging, including transcriptomic and epigenomic variability [40,41,42].

### 2.2. In Vivo Approaches

The generation of mouse models that mimic the disease to the greatest extent possible is an important contribution to the arsenal of research tools for JMML. An ideal animal model would recapitulate salient JMML features such as hypersensitivity of hematopoietic progenitors to GM-CSF, monocytosis, anemia, thrombocytopenia, hepatosplenomegaly, and infiltration of peripheral tissues with leukemic cells [1]. Many efforts to this end have already been made. The earliest studies used mice with genetically engineered deficiency of the *Nf1* gene [43,44], illustrating the central role of Nf1 in regulating the proliferation and survival of hematopoietic progenitor cells in response to various cytokines [44]. Other models used knock-in strategies to manipulate the *Ptpn11* [45,46] or *Kras* [47,48] genes. Many models had limitations in recapitulating the characteristic picture of JMML due to embryonic lethality, non-hematopoietic expression, or the emergence of lymphoma. A recent study directed the expression of *Kras*^G12D^ to multipotent progenitor cells, producing JMML features such as prenatal *Kras*^G12D^ expression, neonatal onset of leukemia, hepatosplenomegaly, and extramedullary organ infiltration [49]. *Kras*^G12D^ progenitor cells showed hypersensitivity to GM-CSF in colony-forming assays, which was reversible by inhibition of the mitogen-activated protein kinase (MAPK) pathway [49]. Due to the heterogeneous nature of JMML driven by distinct Ras pathway mutations, comprehensive modeling will require the generation of numerous mouse models to cover the spectrum of driver mutations relevant to JMML.

A major problem of transgenic animal models lies in the monodimensional pathogenesis of the tumors produced, leading to reduced complexity of the imitated human neoplasia. An alternative approach is the expansion of human leukemia-initiating cells in immunodeficient mice, which lack the capability of xenologous graft rejection. Several papers reported the use of primary cells obtained from JMML patients for xenotransplantation [2,13,50,51,52,53]. It was demonstrated that JMML progenitor cells were capable of initiating the disease in severe combined immunodeficiency mice after direct and serial transplantation [50], confirming their cancer stem cell properties. Engrafted mice were then used to test potential therapies, including the induction of remission in JMML xenograft mice by inhibiting GM-CSF [51]. More recently, our group generated a xenotransplantation model on the background of *Rag2*^–/–^*γc*^–/–^ mice, which was characterized by clonal expansion of myelomonocytic progenitor cells in murine BM, spleen, liver, and lung. Phenotype and engraftment kinetics were similar in secondary and tertiary recipients after serial retransplantation, achieving long-term ex vivo leukemia cell propagation [2]. The model was used to confirm the origin of aberrant epigenetic patterns in leukemia-initiating cells, and to test DNA-hypomethylating therapy for JMML in vivo [2]. Shortcomings of xenotransplantation relate to uneven reproducibility of engraftment and potential loss of valuable sample material due to early death of experimental animals before full engraftment is achieved. Moreover, PB is an unreliable source to monitor engraftment, since PB kinetics only insufficiently reflect the development of leukemia in the BM. In addition, the use of xenotransplantation to model JMML requires animal welfare precautions and involves issues of expense and manpower.

**Figure 1:**
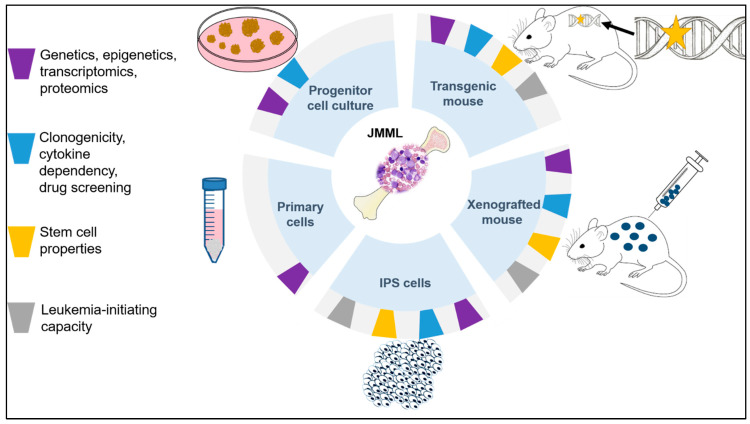
Preclinical modeling of JMML.

Disease models used to investigate JMML are summarized in Figure 1.

## 3. IPSCs as an Emerging Approach to Model Human Disease

Fifteen years after the first successful reprogramming of somatic cells into induced pluripotent stem cells [54,55], a countless number of IPSC lines have been generated [56]. Similar to embryonic stem cells (ESCs), IPSCs possess unlimited self-renewal capacity [54,55], retain the potential to differentiate into cell types of the three germ cell layers [54,55,56,57,58], and can be maintained and expanded in large amounts [56]. IPSC technology has since become more mature and safe, paving the way for the feasible use of personalized cell therapy, regenerative tissue therapy, or drug discovery via large-scale screening. For example, IPSC technology has entirely opened up new insights and experimental possibilities in the field of cardiac dysfunction [59,60,61]. When IPSC-derived cardiomyocytes were used to model the risk of cardiac arrhythmia induced by sotalol, individual changes in cardiac repolarization correlated strongly with those observed clinically in the patients from which each IPSC line was generated [62]. It was also demonstrated that IPSC-derived cardiomyocytes from breast cancer patients at the cellular level reflected the individual susceptibility to doxorubicin-induced impairment of mitochondrial function, calcium handling, and antioxidant activity, and hence the patient-specific risk of developing clinical cardiotoxicity [63].

### 3.1. Methods Used for Reprogramming

Forcing ectopic expression of only four basic transcription factors (POU domain class 5 transcription factor 1 [Pou5f1], sex determining region Y-box 2 [Sox2], myelocytomatosis [Myc], and Kruppel-like factor 4 [Klf4]), Kazutoshi Takahashi and Shinya Yamanaka succeeded in converting differentiated somatic mouse, and later also human, cells into pluripotent stem cells closely resembling ESCs [54,55]. Simultaneously, James Thomson’s group successfully reprogrammed primary human fibroblasts to human IPSCs using POU5F1, KLF4, SOX2, and LIN28 [58]. Since that time, a wide range of viral [64,65,66,67,68,69,70] and nonviral [71,72,73,74,75,76] reprogramming techniques were developed and applied. Almost all variations use at least one of the original Yamanaka transcription factors, notably the pluripotency master regulator Pou5f1/POU5F1. However, some recent studies have revealed that POU5F1 is not indispensable for reprogramming under specific environments or in the presence of small molecules that have the potential to control its endogenous expression [77,78].

According to the genomic integration capacity of each particular system, the reprogramming tools can be divided into integrative (retrovirus, lentivirus, piggyBac transposons) and non-integrative (adenovirus plasmid DNA, minicircle DNA, episomal DNA) [79]. So-called next-generation reprogramming does not use any DNA material, thus ensuring a high degree of safety with an added benefit of better efficiency [80]. Sendai viruses represent the most widespread tool of this new generation, having rapidly advanced to become the de facto standard for straightforward and safe reprogramming.

In 2013, the Mitchell Weiss laboratory in Philadelphia was the first to report the generation of IPSCs from mononuclear BM cells of two JMML patients with somatic heterozygous *PTPN11* p.E76K mutations [18]. To date, various viral reprogramming methods have been applied to patient-derived JMML cells, including STEMCCA lentivirus expressing doxycycline-regulated POU5F1, SOX2, MYC, and KLF4 [18,20], retroviral pMXs-based vectors expressing the factors separately [19,22], and Sendai virus encoding the same transcription factors [21] (Table 1).

### 3.2. Types of Reprogrammed Cells

Theoretically, all of the approximately 220 human somatic cell types are reprogrammable [81,82,83]. Indeed, a vast array of cell types have been reprogrammed successfully [84], including fibroblasts [55,57,85], PB cells [86,87], neuronal progenitor cells [88], keratinocytes [89], B cells [90], T cells [91], and hepatocytes [92]. In order to generate disease models for cancer, several groups reprogrammed different types of primary neoplastic cells including leukemia. Reports comprise the establishment of IPSCs from primary myeloblasts and T cells of patients with acute myeloid leukemia (AML) [93], from BM mononuclear cells of patients with chronic myelogenous leukemia (CML) [94], from BM cells of a T-cell acute lymphoblastic leukemia mouse model [95], from BM mononuclear cells of *PTPN11* and *CBL* JMML patients [18,20], from skin fibroblasts of JMML patients with NS [19,22], and from blood T cells of a healthy individual and a patient with *PTPN11*-mutated JMML [21]. However, it is not uncommon for leukemic cells to be refractory to reprogramming, resulting in a lack of patient-derived IPSCs for many genetic subtypes. In addition, differentiating long-term repopulating hematopoietic cells from IPSCs is a notoriously difficult and inefficient process [96].

## 4. Differentiation of IPSCs to Hematopoietic Cells

Procedures to differentiate IPSCs into various cell types are available in the literature, including cardiomyocytes [62], neurons [97], adipocytes [98], endothelial cells [99], and hematopoietic cells [86,87,88,100,101,102]. In fact, the first in vitro production of hematopoietic cells from human ESC in co-culture with murine stroma cells dates back no less than 20 years [101]. Early protocols for in vitro differentiation of hematopoietic stem cells were mostly based on 2D flat co-culture procedures with or without murine BM-derived OP9 cells [102,103,104]. On the one hand, the feeder cell system is robust and has the advantage of obviating the need to add exogenous cytokines. On the other hand, OP9 cells are sensitive to variation in maintenance environments, including medium source and serum lot, which can disturb their capacity to efficiently support hematopoietic differentiation [102]. The use of classical 2D flat culture systems involves particular restrictions regarding cell expansion, differentiation efficacy and stability of differentiation [105]. However, 3D systems using multicellular embryoid bodies (EBs) are now established and overcome these limitations to a great extent. The spheroid aggregates are easily generated from IPSCs in a suspension system and provide a peculiar physiological microenvironment not achieved by 2D culture systems [106,107]. Using the EB approach, in vitro blood cell differentiation is now easy to accomplish by continuous or intermittent use of a palette of different hematopoietic growth factors [18,19,20,21,22,108,109]. Bone morphogenetic protein 4 (BMP4) modulates the proliferative and differentiative potential of hematopoietic progenitors [110,111,112,113] and promotes hematopoiesis from ESCs [114]. Vascular endothelial growth factor regulates hematopoietic stem cell survival [115]. Stem cell factor (SCF) in combination with TPO, interleukin-6 and interleukin-3 induce the proliferation of megakaryocytic progenitor cells [116,117,118]. FLT-3 ligand stimulates the proliferation of primitive hematopoietic progenitors [119]. Basic fibroblast growth factor positively regulates hematopoiesis by acting on stromal cells and hematopoietic progenitors and antagonizing the inhibitory effects of transforming growth factor beta [120]. Interleukin-11 acts on pluripotent and erythroid progenitors by regulating the early stages of hematopoiesis and many phases of erythropoiesis [121]. Erythropoietin guides multipotent hematopoietic progenitor cells toward an erythroid fate [122]. Activin A enhances the production of hematopoietic cells by promoting the induction of mesoderm in combination with BMP4, SCF, and FLT-3 ligand [123]. Insulin-like growth factor 1 stimulates erythrocytes and lymphocytes [124] and increases hematopoietic progenitor cell cloning efficiency [125]. GM-CSF enhances the differentiation of granulocytes, macrophages, and dendritic cells from hematopoietic progenitor cells [126]. Examples of blood cell differentiation achieved with combinations of these cytokines include B lymphocytes [109], T lymphocytes [127,128], myelomonocytic cells [18,19,20,21,22], natural killer cells [127], and erythroid cells [100,129].

The JMML-derived IPSCs reported so far were differentiated into hematopoietic cells via formation of EB as well through an adherent monolayer culture system. Since the reprogramming efficiency of both systems turned out to be comparable, the subsequent studies were based on the simpler EB system [19,20,21,22]. Interestingly, JMML-IPSC-derived hematopoietic progenitor cells exhibited faster proliferation and higher proportion of myeloid cells compared to those generated from healthy control [18,19,20,21]. Furthermore, another study observed that IPSCs derived from Noonan syndrome (NS)/myeloproliferative disorder (MPD) cells produce a considerably greater number of leukocytes (CD45+), myeloid (CD33+) and erythroid cells (CD235a+) compared to both NS and wild-type cells [22]. Except for two studies [18,21], where CD34+ cells were already detected at day 8, most investigators observed the formation of hematopoietic cells between day 12 and 14 after initiation of differentiation.

Despite well-established procedures, in vitro differentiation protocols are not equally efficient for all cell types [96,130]. Several studies have therefore investigated the ability of IPSCs to generate hematopoietic stem and progenitor cells (HSPCs) under in vivo conditions [131,132]. Human IPSCs gave rise to CD34+CD45+ populations and subsequently to differentiated myeloid and lymphoid progenitors in immunocompromised mice [96,133]. Here, the differentiation protocols were based on the use of teratoma to provide a physiological microenvironment. Furthermore, isolated HSPCs reconstituted a human immune system when transplanted into immunodeficient mice. Subsequently, successful in vivo generation of HSPCs from CRISPR/Cas9-edited IPSCs has been reported [134]. The authors described that IPSC-derived HSPCs have hematopoiesis-reconstituting potential and long-term engraftment capacity. However, despite relative simplicity, low cost, and lack of need for exogenous growth factors, HSPCs obtained from teratomas have not yet been developed for clinical application.

## 5. Role of Preexistent Genetics and Epigenetics on Leukemia Formation

It is expected that reprogramming of somatic cells is accompanied by a thorough reset of the epigenetic landscape, leading to a whole new epigenome. Accordingly, histone acetylation and DNA hypomethylation at regulatory regions of ESC-specific genes were described, whereas the opposite occurs at tissue-specific genes [135,136]. At the same time, IPSCs at low passage still harbor residual DNA methylation patterns of the somatic cell of origin [137]. It is therefore thought that heterogeneous differentiation properties of IPSCs are attributable to incomplete removal of somatic epigenetic marks, beside other factors like chromosomal aberrations or gene mutations [138]. This phenomenon is referred to as “epigenetic memory”. It was suggested that subsequent differentiation and serial reprogramming washes out the epigenetic memory of the starting cells [139,140].

Various epigenomic investigations have highlighted the pivotal role of epigenetic changes in the development and progression of myeloid neoplasia, especially AML [141,142,143,144]. Consistent with the above concept, the reversion of AML cells to IPSCs was accompanied by a universal reset of DNA methylation changes associated with leukemic transformation [93]. It is therefore remarkable that cells differentiated in vivo from AML-IPSCs still had high similarity in DNA methylation and gene expression profiles to the primary leukemic cells [93]. However, it was also noted that the effect of epigenetic memory on the phenotype of cells differentiated from AML-IPSCs was highly dependent on the cell type context [93]. Since one or more genetic driver mutations were maintained all the way through IPSC formation and were required (and sufficient) to recreate the leukemic phenotype, it was concluded that neoplastic transformation to AML, including the epigenetic changes associated with it, results from the activity of specific gene mutations rather than preformed epigenetic alterations [93]. In contrast to AML, IPSCs derived from CML cells are characterized by significantly diverse DNA methylation in the presence of a uniform genetic driver (the *BCR*-*ABL1* fusion oncogene). Despite this “epigenetic drift”, CML-IPSC-derived CD45+/CD34+ cells still retain the potential to acquire myeloid and erythroid differentiation capabilities in vitro and in vivo and can give rise to typical CML phenotypes, including CD15+/CD14+/GlyA+ [145].

The significance of epigenetic profiles during reprogramming and differentiation has not yet been explored in JMML. Given the strong correlation of aberrant DNA methylation with clinical outcomes in this disorder, such experiments appear particularly attractive. They will provide valuable insight as to whether epigenetic memory influences stem cell properties in JMML and identify key epigenetic modifications that accompany transformation to JMML.

## 6. Applications of JMML-Derived IPSCs

It is unsatisfactory that HSCT remains the only curative modality of treatment for most children diagnosed with JMML, given its toxicity and high risk of relapse [146]. Hence, there is an obvious need for preclinical development of new therapy strategies. In light of the limitations of working with primary JMML material discussed above, the use of JMML-derived IPSCs appears to be a viable and promising alternative approach.

Among other intracellular signaling molecules, mitogen-activated protein kinase kinase (MEK) and janus protein tyrosine kinase 1/2 (JAK1/2) are hyperactive in JMML [8,10,11]. Shilpa Gandre–Babbe and colleagues showed higher sensitivity of JMML-derived IPSCs to the MEK and JAK1/2 kinase inhibitors PD901 and ruxolitinib than IPSCs derived from healthy control cells [18]. Reduced colony-forming activity of JMML progenitors, diminished colony size, normalized response to GM-CSF, and inhibition of cytokine-independent formation of myelomonocyte colonies were in line with other studies in *Nf1*- and *Kras*-mutant mice, where JMML-like MPDs were attenuated by treatment with PD901 [147,148]. By contrast, the effect of ruxolitinib was rather weak, suggesting a minor role of JAK1/2-mediated signal transduction in *PTPN11*-mutated JMML [18].

Aiming to identify targetable effector molecules within the Ras kinase cascade, Sarah Tasian and colleagues generated IPSCs from *PTPN11*- and *CBL*-mutant JMML, characterized the differences between their signaling profiles, and examined the effect of various kinase inhibitors [20]. The authors compared the size of myeloid colonies differentiated from the two mutant IPSC types and control IPSCs and found that colonies originating from either mutation were larger and more dispersed than controls. However, there was no difference in colony formation and sensitivity to GM-CSF between the two mutants. Phosphoflow cytometry was then used to describe distinct signaling profiles for both mutational categories. The Ras/MAPK signaling pathway was hyperactive in *PTPN11*-mutant IPSCs compared to *CBL* mutants, and vice versa for the JAK/signal transducer and activator of transcription (STAT) axis [20]. Different kinase inhibitors were then employed to target these specific profiles. The analysis showed superior efficacy of the MEK inhibitors PD0325901 and trametinib in *PTPN11*-mutant cells. Conversely, the JAK/STAT inhibitors momelotinib and ruxolitinib were more effective in cells with a *CBL* mutation [20]. The comparison of the phosphoinositide 3-kinase delta (PI3K δ) inhibitor idelalisib and the mammalian target of rapamycin (mTOR) inhibitor rapamycin showed that both categories of JMML IPSCs responded similarly to these agents. The authors advocated kinase inhibitors as potential candidates for further drug development in JMML. Moreover, they suggested that rapamycin might be promising as a general therapy principle in JMML, independently of the particular driver mutation [20].

A more recent study assessed the proteome of myeloid cells derived from IPSCs of one healthy individual (wildtype, WT), one patient with NS and MPD, and one patient with NS but no MPD [22]. The authors detected significant differences between the proteomes of WT and both NS and NS/MPD, as well as between NS and NS/MPD. The expression of integrin beta 2 and the calcium-binding protein S100-A4 was markedly increased in NS compared to WT and even more increased in NS/MPD. In addition, the expression of the tumor suppressor protein TP53 was decreased in both NS and NS/MPD-derived myeloid cells, and the expression of the nuclear factor kappa light chain enhancer of activated B cells (NF-κb) inhibitor was increased in both NS and NS/MPD [22].

These results, including a transcriptome analysis highlighting the proto-oncogene *MYC* as a key regulator [19], suggested an important role of the MYC, TP53, and NF-κb signaling pathways for altered protein expression in JMML. To assess the therapeutic exploitability of these pathways, three drugs were used on NS and NS/MPD IPSCs- derived myeloid cells: CBL0137, which inhibits NF-κb; JQ1, which inhibits MYC; and Nutlin, which destabilizes TP53 [149]. All three drugs had an effect, which was strongest in the case of CBL0137. Specifically, the colony-forming capacity of NS and NS/MPD cells decreased significantly with exclusive formation of myeloid colonies and the absence of erythroid colonies [22].

Upregulation of micro-RNAs miR-223 and miR-15a was observed in myeloid cells differentiated from IPSCs after reprogramming fibroblasts of 2 patients with germline *PTPN11* mutation [19]. This upregulation correlates with in vivo findings in primary BM mononuclear cells of patients with germline or somatic *PTPN11* mutations. However, the increase in micro-RNA levels was heterogeneous among JMML patients with different mutations. In addition, the genes coding for Ras-related protein Rab-12 and forkhead box O3 were confirmed as bona fide targets of miR-223 by in vitro transcriptional regulation studies using modified HEK293 cells [19].

The somatic tissue of origin deserves careful consideration when embarking on IPSC experiments [150]. With respect to JMML, the field is too immature to compare IPSC phenotypes depending on source cells. In each of the studies cited above, only one tissue of origin was used, and side-by-side comparisons were not made. Other variations, such as the driver mutation and the reprogramming kit, come into play. None of the studies recorded epigenomic JMML IPSC data. At least, it is noteworthy that hematopoietic differentiation of JMML IPSC modeled characteristic features of JMML (such as GM-CSF-hypersensitive progenitor cells and expansion of the myeloid lineage) irrespective of whether IPSC were derived from BM/PB [18,20,21] or fibroblasts [19,22].

Overall, studies to date demonstrate that JMML-derived IPSCs imitate several elementary cell biological properties of JMML (expansion of the progenitor cell compartment, proliferation, response to cytokines). Functionally, these properties can essentially be linked with the Ras-deregulating effect of the driver mutation. To what extent the gene transcription patterns, protein expression profiles, and epigenetic landscape in JMML-derived IPSCs also correspond to those of the original cells remains to be investigated.

## 7. Outlook: The Future of IPSCs in JMML Research

To date, at least five groups succeeded in reprogramming *PTPN11*-mutant patient-derived JMML cells to IPSCs, and then re-differentiating these into JMML-like cells [18,19,20,21,22]. Encouragingly, the JMML-like cells generated in these experiments recapitulated central features of JMML primary cells, including increased formation of myeloid colonies and hypersensitivity to low doses of GM-CSF.

It will now be of interest to also model those JMML subtypes in IPSCs that are driven by the other classic Ras pathway genes: *KRAS*, *NRAS*, and *NF1*. Their systematic evaluation will permit side-to-side comparisons of oncogenic driver capacity not previously possible with conventional progenitor cell cultures or ex vivo cell lysates. In addition, global genetic profiling of JMML has uncovered secondary mutational events (including *EZH2*, *ASXL1*, *SETBP1*, *JAK3*, and others) which correlate with a higher risk of progression and poor clinical outcome [1,8,10,11]. IPSC technology forms an unprecedented basis for gene editing experiments that imitate such secondary mutations in the environment of a faithful cellular JMML model. Conversely, the correction of mutant *PTPN11* in JMML-IPSCs via gene editing was shown to decrease the output of CD34+ hematopoietic progenitor cells to a level comparable with WT-IPSCs [21]. With a little imagination, these and similar achievements can be viewed as prototypes for future targeted and personalized therapy of JMML.

## Figures and Tables

**Table 1:** Summary of the literature on JMML-derived IPSC studies. Abbreviations: HSC, hematopoietic stem cell; MNC, mononuclear cell; BM, bone marrow; PB, peripheral blood; IPSC, induced pluripotent stem cell; JMML, juvenile myelomonocytic leukemia; GM-CSF, granulocyte-macrophage colony-stimulating factor; NS, Noonan syndrome; MPD, myeloproliferative disorder.

Study	Reprogrammed Cells	Mutations	Reprogramming Technique	HSC Differentiation Protocol	Cytokine Combination	Remarks	Reference
Gandre–Babbe et al., 2013	Ficoll-purified MNCs from BM or PB *n* = 2	Somatic heterozygous missense mutations in *PTPN11*	STEMCA lentivirus expressing doxycycline-regulated POU5F1, SOX2, MYC, and KLF4	Embryoid bodies and adherent monolayer culture with supplementation of cytokines	■ 25 ng/mL BMP4 ■ 50 ng/mL VEGF ■ 50 ng/mL SCF ■ 50 ng/mL TPO ■ 50 ng/mL Flt3 ■ 20 ng/mL bFGF ■ 10 ng/mL IL-3 ■ 5 ng/mL IL-11 ■ 2 U/mL EPO ■ 25 ng/mL IGF-1	First generation of IPSCs from JMML cells. In vitro differentiation of JMML-IPSCs produced myeloid cells with leukemic features including high proliferative capacity, activation of GM-CSF, and enhanced STAT5/ERK phosphorylation. The inhibition of MEK kinase in IPSC-derived JMML cells reduced their GM-CSF hypersensitivity	[18]
Mulero–Navarro et al., 2015	Skin fibroblasts *n* = 2	Germline mutations causing NS/MPD	Separate retroviruses expressing human POU5F1, SOX2, MYC, and KLF4	Embryoid bodies and cytokine supplementation	■ 25 ng/mL BMP4 ■ 50 ng/mL VEGF ■ 50 ng/mL SCF ■ 50 ng/mL TPO ■ 50 ng/mL Flt3 ■ 20 ng/mL bFGF ■ 10 ng/mL IL-3 ■ 5 ng/mL IL-11 ■ 2 U/mL EPO ■ 25 ng/mL IGF-1	NS/MPD-IPSC-derived myeloid cells carrying a *PTPN11* mutation exhibited an upregulation of miR-223 and miR-15a, similar to BM mononuclear cells harboring *PTPN11* mutations. Normal myelogenesis was reestablished via reducing miR-223’s function in NS/MPD IPSCs	[19]
Tasian et al., 2019	Ficoll-purified MNCs from BM or PB *n* = 2	Germline *CBL*, somatic *PTPN11*	STEMCA lentivirus expressing doxycycline-regulated POU5F1, SOX2, MYC, and KLF4	Embryoid bodies and cytokine supplementation	■ 25 ng/mL BMP4 ■ 50 ng/mL VEGF ■ 50 ng/mL SCF ■ 50 ng/mL TPO ■ 50 ng/mL Flt3 ■ 20 ng/mL bFGF ■ 10 ng/mL IL-3 ■ 10 ng/mL GM-CSF	MEK, JAK, and PI3K/mTOR inhibitors resulted in different reactivity of IPSC-derived hematopoietic progenitors and signaling aberrations, depending on the driver mutation	[20]
Shigemura et al., 2019	PB T cells *n* = 1	*PTPN11*	Sendai virus vector encoding the human transcription factors POU5F1, SOX2, MYC, and KLF4	Co-culture system with stroma cells	■ 40 ng/mL BMP4 ■ 40 ng/mL VEGF ■ 50 ng/mL SCF ■ 10 ng/mL TPO	Mutant IPSC colonies generated significantly more CD34+ and CD34+ CD45+ cells compared to non-mutant IPSC colonies. The *PTPN11* mutation seems to govern hematopoietic differentiation in JMML	[21]
Pearson et al., 2020	Skin fibroblasts *n* = 2	NS/MPD	Separate retroviruses expressing human POU5F1, SOX2, MYC, and KLF4	Embryoid bodies and cytokines supplementation	■ 20 ng/mL BMP4 ■ 10 ng/mL bFGF ■ 5 ng/mL activin A ■ 10 ng/mL bFGF and VEGF ■ 25 ng/mL insulin growth factor-1 ■ 2 U/mL EPO ■ 10 ng/mL interleukin-11, bFGF, VEGF, interleukin-3, interleukin-6 ■ 50 ng/mL SCF, TPO	Establishment of proteomic screen in NS-derived IPSCs. Demonstration of additive effects of two drugs (JQ1 as differentiation enhancer and CBL0137 as apoptosis inducer) on NS/MPD cells.	[22]

## Data Availability

Not applicable.
